# Down-regulation of IRES containing 5'UTR of HCV genotype 3a using siRNAs

**DOI:** 10.1186/1743-422X-8-221

**Published:** 2011-05-13

**Authors:** Saba Khaliq, Shah Jahan, Asim Pervaiz, Usman Ali Ashfaq, Sajida Hassan

**Affiliations:** 1 Applied and Functional Genomics Lab, Centre of Excellence in Molecular Biology, University of the Punjab. Lahore, Pakistan

## Abstract

**Background:**

Hepatitis C virus (HCV) is a major causative agent of liver associated diseases leading to the development of hepatocellular carcinoma (HCC) all over the world and genotype-3a responsible for most of the cases in Pakistan. Due to the limited efficiency of current chemotherapy of interferon-α (IFN-α) and ribavirin against HCV infection alternative options are desperately needed out of which the recently discovered RNAi represent a powerful silencing approach for molecular therapeutics through a sequence-specific RNA degradation process to silence virus infection or replication. HCV translation is mediated by a highly conserved internal ribosome entry site (IRES) within the 5'UTR region making it a relevant target for new drug development.

**Materials and methods:**

The present study was proposed to assess and explore the possibility of HCV silencing using siRNA targeting 5'UTR. For this analysis full length HCV 5'UTR of HCV-3a (pCR3.1/5'UTR) was tagged with GFP protein for *in vitro *analysis in Huh-7 cells. siRNA targeting 5'UTR were designed, and tested against constructed vector in Huh-7 cell line both at RNA and Protein levels. Furthermore, the effect of these siRNAs was confirmed in HCV-3a serum infected Huh-7 cell line.

**Results:**

The expression of 5'UTR-GFP was dramatically reduced both at mRNA and protein levels as compared with Mock transfected and control siRNAs treated cells using siRNAs against IRES of HCV-3a genotype. The potential of siRNAs specificity to inhibit HCV-3a replication in serum-infected Huh-7 cells was also investigated; upon treatment with siRNAs a significant decrease in HCV viral copy number and protein expression was observed.

**Conclusions:**

Overall, the present work of siRNAs against HCV 5'UTR inhibits HCV-3a expression and represents effective future therapeutic opportunities against HCV-3a genotype.

## Background

A large number of people die each year from liver failure and cancer caused by HCV infection as more than 3% world population is chronically infected with this viral pathogen especially in developing countries including Pakistan where 6% of population is infected [1,2]. In 40-60% of HCV infected individuals persistent infection is mainly associated with liver cirrhosis and steatosis leading to HCC [3,4]. The standard treatment for HCV, a combination therapy of pegylated interferon α (PEG-IFN-α) and guanosine analog ribavirin, has limited efficiency, significant expense, poor tolerability and assure long term eradication of the virus in less than half proportion of treated patients largely dependent on the significant variation among different HCV genotypes [5]. In Pakistan, about 75% of patients have no therapeutic benefit to current therapy and approximately 20% of patients have to discontinue therapy due to adverse side effects [6,7]. In Pakistan the major HCV genotype is 3a followed by 3b and 1a with a strong correlation between chronic HCV infection and HCC with genotype 3a [8,9]. Due to the limitations of current therapy the development of better tolerated therapeutic option for HCV is a major objective of the present era. Currently research is focused on exploiting new viral drug targets such as sequence-specific and endogenous mechanism of gene silencing like RNA interference (RNAi) for human therapy and gene function studies.

RNAi, a recently described phenomenon in which post-transcriptional regulation of protein expression, is done by small double stranded RNA (dsRNA), called as siRNA, inducing sequence-specific degradation of homologous target mRNA recognized by antisense strand of siRNA [10-16]. siRNAs can be used as potential therapeutic agent against HCV, because HCV replication takes place in cytoplasm of liver cells, primary target, without integration into host genome. Moreover, its genome functions both as mRNA and a replication template. So the destruction of HCV RNA could eliminate not only protein synthesis but also viral replication. siRNA directed against the viral genes including 5'untranslated region (5'UTR) of HCV 1a, 1b and 3a genotype (recently by our group) effectively blocked the replication of viral replicons in Huh-7 derived cell lines [17-30]. The development of siRNA targeted to 5'UTR of local genotype 3a which are crucial for initiation of viral translation provides better options for developing a rational antiviral strategy against this local HCV genotype.

HCV is a positive single-stranded RNA (ssRNA) enveloped virus approximately 9.6 kb in length with an open reading frame (ORF) encoding a large viral polyprotein of about 3010 amino acids [31,32]. Viral translation is mediated through an internal ribosome entry site (IRES) found within the 5'UTR. The sequence of 5'UTR ~341 bp in length is highly conserved even between different HCV isolates. 5'UTR does not encode for functional protein and contains IRES that initiate translation of the viral polyprotein in a cap-independent manner. The IRES has a key role in translational events as it binds independently to the 40S ribosomal subunit and directs the ribosome to the initiation codon of the HCV mRNA in order to facilitate translation in a cap-independent manner [33,34]. It contains four highly structured stem-lopped domains (domain I-IV) that facilitate the translation of HCV RNA [35,36]. Domain I is not required for IRES activity but essential for HCV replication, IRES in Domain II-IV mediates the cap independent translation of viral genes [35,37,38]. Domain III contains subdomains which are essential for the binding of 40S ribosomal subunit [39].

Viral escape and off-target effects due to RNA silencing is a major problem in development of effective RNAi based antiviral therapy but that can be overcome by finding highly effective target sites. Huh-7 cells are highly permissive for HCV replication and are widely used for the study of HCV-associated diseases and antiviral strategies like RNAi. The present study was undertaken to study the effect of HCV genotype 3a 5'UTR specific siRNAs using a tagged mammalian expression vectors of HCV 5'UTR-GFP and potential of these siRNAs in reduction of viral titer in serum-infected Huh-7 cells.

## Materials and methods

### Source of samples

The local HCV-3a patient's serum samples used in this study were obtained from the CAMB (Center for Applied Molecular Biology) diagnostic laboratory, Lahore, Pakistan after quantification and genotype assessment. Serum samples were stored at -80°C prior to RNA extraction for cloning and viral inoculation experiments. Patient's written consent and approval for this study was obtained from institutional ethics committee.

### Plasmid construction and siRNA designing

For the construction of expression plasmid, viral RNA was isolated from 100 μl serum aliquots using Gentra RNA isolation kit (Gentra System Pennsylvania, USA) according to the manufacturer's instructions. 100-200 ng extracted viral RNA was used for RT-PCR using the SuperScript III one step RT-PCR system (Invitrogen Life technologies, USA). HCV complementary DNA (cDNA) of full length 5'UTR was amplified using specific primers; 5'UTR-F 5'GCAAGCTTACCTGCCTCTTACGAGGC'3 and 5'UTR-R 5'AAGATATCGTTGCACGGTCTACG'3. After amplification, PCR product was tagged with GFP gene, as 5'UTR contain IRES and does not encode any protein, isolated from pUbC-GFP vector. The ligated gene product was digested along with pCR3.1 vector (kindly provided by Dr. Zafar Nawaz, University of Miami, USA) employing primers with *EcoRV *and *XbaI *restriction sites.

To express RNAi mechanism against 5'UTR region of HCV-3a genome, siRNA oligonucleotides were designed using the Ambion's siRNA design tool http://www.ambion.com/techlib/misc/siRNA_finder.html. The designed siRNAs (HCV-3a 5'UTR and control scrambled siRNA) were synthesized using Silencer siRNA construction kit according to the manufacturer's instruction (Ambion, USA). Negative control siRNA (scrambled siRNA) with the same nucleotide composition as the experimental siRNA but lacks significant sequence homology to the HCV and human genome was design (Table 1).

**Table 1:** Sequence of siRNA oligonucleotides directed against 5'UTR of HCV 3a genotype

No.	siRNA name	Sequences 5'-3'
1	Usi170- antisense	AATCGCTGGGGTGACCGGGTCCCTGTCTC
2	Usi170-sense	AAGACCCGGTCACCCCAGCGACCTGTCTC
3	Usi212- antisense	AATACCCAGAAATTTGGGCGTCCTGTCTC
4	Usi212- sense	AAACGCCCAAATTTCTGGGTACCTGTCTC
5	Usi272- antisense	AAAGGCCTTGTGGTACTGCCTCCTGTCTC
6	Usi272- sense	AAAGGCAGTACCACAAGGCCTCCTGTCTC
7	Sc-antisense	AACCTGCATACGCGACTCGACCCTGTCTC
8	Sc-sense	AAGTCGAGTCGCGTATGCAGGCCTGTCTC

### Cell culture and transfection

Huh-7 cell line was kindly provided by Dr. Zafar Nawaz (University of Miami, USA) and routinely maintained in Dulbecco's modified eagle medium (DMEM) supplemented with 100 μg/ml penicillin:streptomycin and 10% fetal bovine serum (Sigma Aldrich, USA) at 37°C with 5% CO_2_. To examine the effects of HCV-3a 5'UTR siRNAs, Huh-7 cells were transfected with specific or scrambled siRNAs along with HCV-3a 5'UTR-GFP vector. Briefly, cells were seeded in 24-well (1 × 10^5^/well) or 6-well (5 × 10^5^/well) plates and cultured in complete medium until they became 60-80% confluent. Cells in 24-well plates were transiently transfected with 10, 20, 40 nM/well of specific siRNAs or scrambled siRNA along with 0.4 μg of HCV-3a constructed vectors in serum free media using Lipofectamine™ 2000 (Invitrogen Life technologies, CA) according to the manufacturer's protocol. After 6 hrs incubation at 37°C in 5% CO_2_, complete medium was added to the cells. Protein analysis was carried out for above mentioned experiments in 6-well plates with 100 μM/well of each siRNA. Cells were harvested at 24 and 48 hrs post-transfection for gene expression analysis.

### Isolation of total RNA and gene expression

Total RNA from transfected and non-transfected cells was isolated using TRIzol reagent (Invitrogen life technologies, CA) 24 hrs and 48 hrs post-transfection. To analyze the effect of siRNA, cDNA was synthesized with 1 μg of total isolated RNA, using Superscript III cDNA synthesis kit (Invitrogen life technologies, CA) and semi-quantitative RT-PCR was done using primers of 5'UTR and GAPDH as control. Quantitative Real Time PCR was carried out using Real Time ABI 7500 system (Applied Biosystems Inc, USA) with SYBR Green mix (Fermentas International Inc, Canada) using gene specific primers 5'UTR-F 5'TCACTCCCCTGTGAGGAACT'3, 5'UTR-R 5'TCCCGGGGCACTCGCAAGCA'3. GAPDH gene was used for normalization as control using GAPDH-F 5'ACCACAGTCCATGCCATCAC'3 and GAPDH-R 5'TCCACCACCCTGTTGCTGTA'3. The relative gene expression analysis was carried out by the SDS 3.1 software (Applied Biosystems Inc, USA). Each individual experiment was performed in triplicate.

### Western Blotting

To determine the protein expression levels of GFP gene under the control of 5'UTR, the transfected and non-transfected (with and without siRNAs) cells were lysed with ProteoJET mammalian cell lysis reagent (Fermentas, Canada). Equal amounts of total proteins were subjected to electrophoresis on 12% SDS-PAGE and electrophoretically transferred to a nitrocellulose membrane following the manufacturer's protocol (Bio-Rad, CA). After blocking non-specific binding sites with 5% skimmed milk, blots were incubated with primary monoclonal antibodies specific to GFP, Core, E1, E2 and GAPDH (Santa Cruz Biotechnology Inc, USA) and secondary Horseradish peroxidase-conjugated anti-goat anti-mouse antibody (Sigma Aldrich, USA). The protein expressions were evaluated using chemiluminescence's detection kit (Sigma Aldrich, USA).

### Viral inoculation and co-transfection with siRNA

Huh-7 cell line was used to establish the in vitro replication of HCV. A similar protocol was used for viral inoculation as described previously [28,40]. Briefly, high viral titer > 1 × 10^8 ^IU/ml from HCV-3a patient's sera was used as principle inoculum in these experiments. Huh-7 cells were maintained in 6-well culture plates to semi-confluence, washed twice with serum-free medium, and then inoculated with 5 × 10^7^IU/well viral load of HCV-3a sera and 500 μl serum free media. Cells were maintained overnight at 37°C in 5% CO_2_. Next day, adherent cells were washed three times with 1× PBS, complete medium was added and incubation was continued for 48 hrs. Cells harvested and were assessed for viral RNA quantitatively by Real Time PCR. To analyze the effect of siRNA on HCV infection, serum infected Huh-7 cells were again seeded after three days of infection in 24-well plates and grown to 80% confluence with 2 ml medium. The cells were transfected with or without 40 μM/well of siRNAs using Lipofectamine™ 2000 (Invitrogen Life technologies, CA) according to the manufacturer's protocol.

### Viral Load

Cells were harvested for intracellular viral RNA determination using Gentra RNA isolation kit (Gentra System Pennsylvania, USA) according to the manufacturer's instructions. For viral quantification (assay based on the detection of 5'UTR) of viral copies) Sacace HCV quantitative analysis kit (Sacace Biotechnologies Caserta, Italy) was used. Briefly, 10 μl of extracted viral RNA was mixed with an internal control derived from 5'UTR provided by Sacace HCV Real TM Quant kit and subjected to viral quantification using Real Time PCR SmartCycler II system (Cepheid Sunnyvale, USA). For viral protein expression analysis at day 3 of viral inoculation, cells were washed 3 times before harvesting and Western blotting was performed.

### Statistical Analysis

All statistical analysis was done using SPSS software (version 16.0, SPSS Inc). Data are presented as mean ± SD. Numerical data were analyzed using student's t-test and ANOVA. P value < 0.05 was considered statistically significant.

## Results

### Screening of siRNAs against HCV 5'UTR at RNA level

**Figure 1:**
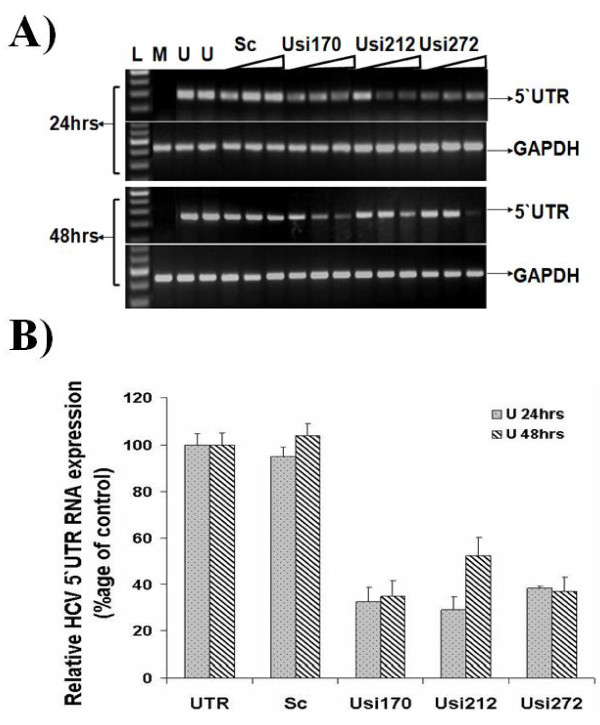
**HCV 3a 5'UTR specific siRNAs inhibit mRNA expression**. **A) **Huh-7 cells were transfected with 0.4 μg of constructed HCV vector or mock-treated along with or without 10 nM, 20 nM and 40 nM of siRNAs for 24 and 48 hrs. Cells were harvested and relative RNA determinations were carried out using semi-quantitative PCR. Gene expression results from semi-quantitative PCR are given for increasing concentrations of Usi170, Usi212 and Usi272 siRNAs against HCV 3a 5'UTR. Expression levels for mock-transfected (M), HCV 3a 5'UTR expression plasmid (U), scramble siRNA (Sc), 100 bp DNA Ladder (L) and GAPDH are also shown. **B) **Huh-7 cells were transfected pCR3.1/GFP/5'UTR vector or mock-treated along with or without 40 nM of siRNAs for 24 and 48 hrs. Total cellular RNA extracted, after 24 and 48 hrs post transfection, was quantified by Real Time PCR using gene specific primers in comparison to Mock. Gene expression results from Real Time PCR shows that Usi170 and Usi272 siRNAs against HCV 3a 5'UTR decrease RNA expression both after 24 and 48 hrs post transfection. GAPDH was used as internal control. Three independent experiments were performed having triplicate samples. Error bars indicate mean S.D, p < 0.01.

Due to highly conserved region of HCV is indispensible for both RNA translation and replication 5'UTR has been a focus of antiviral research, therefore in the present study we designed and tested siRNAs targeting 5'UTR of HCV-3a to evaluate their antiviral activity against important domains for translation initiation. To test the HCV 3a directed siRNAs for their ability to suppress the well conserved HCV IRES (5'UTR) mediated translation, three siRNAs were designed to trigger RNAi and co-transfected with constructed vector into Huh-7 cells in different concentrations. Huh-7 cells were transfected with pCR3.1/GFP/5'UTR, that expresses GFP under the control of HCV 5'UTR, with and without 5'UTR specific siRNAs. All siRNAs inhibited the expression of GFP gene in a dose dependent manner (10, 20 and 40 nM) compared with control siRNA. The inhibitory effect of Usi170 siRNA against 5'UTR is stronger at 24 hrs and 48 hrs post transfection even at low concentration, while Usi212 and Usi272 shows more effect after 24 and 48 hrs transfection at 40 nM compared with scramble siRNA which showed no significant change in expression of transfected vector to control cells. In all the siRNA screening experiments, treatment with synthetic and control siRNA did not affect the levels of cellular gene GAPDH expression (Figure 1A). The results of relative quantitative analysis revealed that the transcript levels of the HCV 5'UTR were decreased to 67% in treated cells with Usi170, 71% with Usi212 and 62% with Usi272 at 24 hrs post-transfection, while 65% with Usi170, 48% with Usi212 and 36.7% with Usi272 at 48 hrs post-transfection. A maximum inhibition of 60-70% was observed with Usi170. GAPDH transcript levels showed no change in non-transfected or transfected cells (Figure 1B).

**Figure 2:**
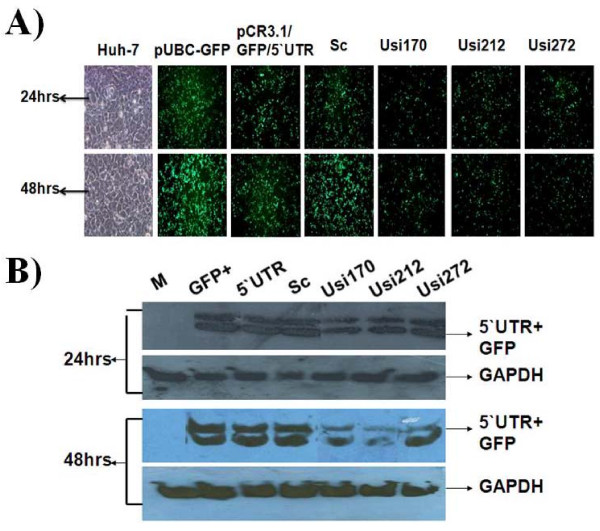
**HCV-3a 5'UTR-specific siRNA inhibit GFP protein expression**. **A) **Silencing effect of HCV-3a 5'UTR- siRNA on 5'UTR-GFP tagged protein. GFP fluorescence was observed under illumination with 360-400 nm light that excites GFP fluorescence (20× magnification) with and without siRNA tranfected Huh-7 cells. **B) **Silencing of HCV-3a 5'UTR-GFP gene by siRNAs using GFP specific antibodies show reduction at protein expression level. The protein expression levels were determined by Western blot analysis after 24 and 48 hrs transfection with mock (M), HCV 3a 5'UTR expression plasmid (U), with and without HCV 3a siRNAs (Usi170, Usi212 and Usi272) and scramble siRNA (Sc) in Huh-7 cells. Protein levels for GAPDH gene are also shown as internal control.

At 24 and 48 hrs post-transfection, GFP expression in Huh-7 cells was also photographed under a fluorescent microscope; the results suggested that GFP fluorescence was decreased in siRNA transfected cells as compared to pUbC-GFP as a control vector, +ve control without siRNA and scrambled siRNAs transfected cells. To determine whether changes in GFP fluorescence accurately reflect underlying changes in GFP mRNA presence as a result of RNAi triggered siRNAs, the protein extracts derived from the siRNA transfected cells were tested for the expression of GAPDH and found to be similar in all Huh-7 cells, non-transfected and transfected with HCV plasmid. Interestingly, western blotting showed that GFP protein expression was efficiently inhibited by 65% in cells co-transfected with pCR3.1/GFP/5'UTR, and siRNAs targeting 5'UTR, but not in the cells co-transfected with positive vector and scramble siRNA after 24 and 48 hrs transfection. Moreover, Usi212 was found to be more effective than Usi170 and Usi272 (Figure 2). This data suggest that siRNA not only has a negative effect on HCV-RNA but also it could decrease protein production under the control of 5'UTR.

### Effect of siRNAs against HCV virus in serum infected Huh-7 cells

**Figure 3:**
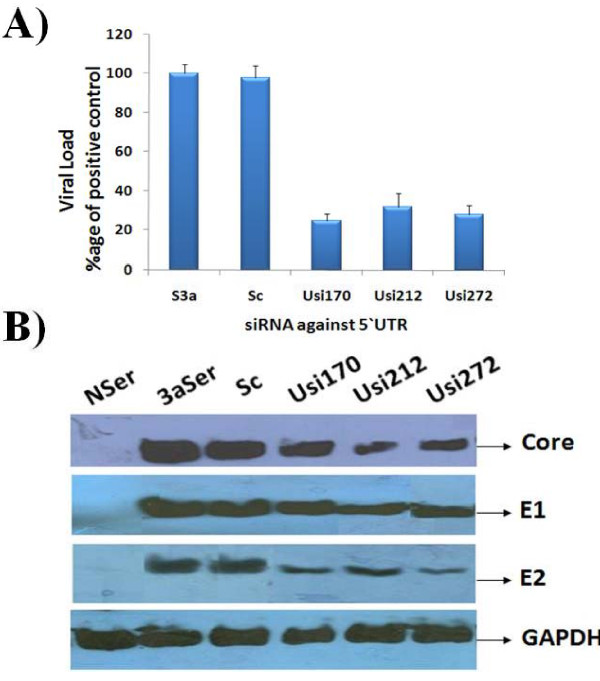
**Silencing effect of HCV 3a genes-specific siRNAs in Huh-7 cells infected with HCV 3a sera**. After 24 hrs post plating, Huh-7 cells were transfected with siRNAs against HCV-3a genes alone and incubated. Next day, cells were washed 5 times with 1XPBS and incubation was continued for additional 48 hrs. Cells were harvested and HCV RNA levels were quantified by Real Time PCR. **A) **Effect of 5'UTR specific siRNAs against HCV. **B) **On 3^rd ^day of infection cells were harvested and protein was subjected to SDS PAGE and expression levels were determined by Western blot analysis. In the figure Normal serum infected cells (Nser), HCV-3a serum infected cells (Ser3a), Cells infected with scrambled siRNA (Sc), Gene specific-siRNA against 5'UTR. Data are expressed as mean percent viral load of non-siRNA treated samples. Three independent experiments with triplicate determinations were performed. Error bars indicate, mean S.D p < 0.05 verses S3a.

The present study was undertaken to design and test siRNA as an alternative therapy against HCV, the results against constructed vector indicate that siRNA targeting HCV 5'UTR has the ability to inhibit the replication of HCV RNA and protein in Huh-7 cells. Taken advantage of the newly developed cell culture system of HCV infection in liver cells [27,28,41], the effect of siRNAs against whole virus was evaluated by treating cells with siRNA. Huh-7 serum infected cells were treated with 5'UTR siRNAs and subsequently incubated for 3 days. Total cellular RNA was extracted from Huh-7 cells and Real Time PCR was performed with 5'UTR specific primers to analyze the down regulation of HCV RNA by gene specific siRNAs. Maximal inhibition of HCV transcript levels was detected on Day 3 post transfection, the results show an approximate 70-80% decrease in HCV RNA levels in HCV serum infected Huh-7 cells that were incubated with different gene specific-siRNAs. This result was in accordance with Zekri et al., [27] result who also showed that best inhibitory effect of siRNAs against 5'UTR on 3^rd ^day of post transfection. No significant inhibition was detected in cells transfected with the negative control siRNA. siRNAs targeting IRES containing 5'UTR showed upto 80% inhibition of viral load. Taken together a significant level of HCV genotype 3a viral RNA suppression by siRNA against 5'UTR was observed and these data suggest a negative impact of gene-specific siRNAs on HCV replication (Figure 3A). Moreover, siRNAs effect on HCV proteins (Core, E1, and E2) in serum infected Huh-7 cells was detected using gene specific antibodies. Densitometric analysis revealed no significant inhibition at protein levels in cells transfected with the negative control siRNA (scrambled siRNA). Two siRNAs targeting IRES containing 5'UTR (Usi170 and Usi272) showed upto 60-70% inhibition of viral proteins expression especially E2 and upto 40% inhibition of Core protein expression (Figure 3B). Together, the data suggest a negative impact of chemically synthesized siRNA against 5'UTR on the down regulation of HCV genes activity as a target for preventing HCV induced HCC development. These results are in accordance with our previous data in which Core and envelope genes were targeted by siRNAs which had a inhibitory effect on total viral titre [28].

## Discussion

As a gene silencing mechanism, RNAi represents an exciting technology with potential applications for treatment of viral diseases such as HCV. Several laboratories have demonstrated that RNAi efficiently inhibits viral replication and expression of HCV genotype 1a and 1b using replicon cell lines by targeted 5'UTR, Core, E2, NS3, NS4b and NS5b sequences by using RNAi [42]. Recently our group tested siRNAs against structural genes of HCV genotype 3a which is the most prevalent in Pakistan, showing upto 80% inhibition at RNA and protein levels [28]. A potential problem that may arise in RNAi based approach is the error prone nature of HCV genome but this problem can be overcome by designing siRNAs against highly conserved region like HCV 5'UTR. The IRES containing 5'UTR is the most highly conserved region of HCV genome with more than 90% identity between the sequences of distantly related strains [23,43]. It was proposed that RNAi-based antiviral strategy could be used to degrade 5'UTR of HCV 3a genotype. Given this scenario in the present study, RNAi was utilized to determine the efficacy of this technique for inhibition of HCV 3a 5'UTR as an alternative therapeutic option.

The IRES containing conserved 5'UTR is required for HCV translation which displays greatest homology between different genotypes and has been targeted in many studies with different approaches. 5'UTR have been found effective targets, with inhibition of IRES-dependent translation in replicon systems reaching almost 80% in some cases against HCV 1a and 1b genotypes [19,23,26,30], suggesting a considerable potential of this approach with respect to HCV inactivation. Korf *et al*., [44] reported siRNAs against domain II and III of 5'UTR substantially inhibited subgenomic HCV replication driven by HCV IRES. In the present study, all siRNAs designed targeting 5'UTR were located in the Domain III which is thought to be important in 40S ribosomal subunit binding and initiation of translation. It has been reported that Core protein binds to viral genomic RNA via interaction with Domain III of 5'UTR during viral replication [45,46]. By considering these facts, we designed and tested siRNA against these regions. Huh-7 cells are most suitable for virus replication and other studies; Chemically synthesized siRNAs can be introduced into cells when formulated with lipophilic reagents [47-50], we used Huh-7 cells to study siRNA effect. siRNAs against 5'UTR of HCV 3a with the GFP gene as a reporter reduced the RNA of 5'UTR and GFP in a dose dependent manner upto 75% to 80% with Usi212 being the most effective upto 80% inhibition (Figure 1). The effect of gene silencing was evident even after 24 hrs post siRNA transfection in our study and therefore, demonstrating the ability of *in vitro *synthesized siRNAs against silencing the target gene expression.

Guha *et al. *[51] reported that *in vitro *cell culture models can at best demonstrate the infectivity of the virus and used in evaluating drugs for antiviral activity or inhibition of HCV infection. Most of the studies all over the world are conducted in Huh-7 derived cell lines and with replicons supporting HCV RNA transcription and protein synthesis. Recently different groups have studied the HCV replication in serum infected liver cell lines for the study of different HCV genotypes which mimics the naturally occurring HCV virions biology and kinetics of HCV infection in humans [28,52-54]. We infected Huh-7 cells with native viral particles from HCV 3a positive serum, the most prevalent type in Pakistan using the same protocol as established [27,28]. The siRNAs against 5'UTR in the present study were further screened against HCV in serum infected Huh-7 cells. An exciting finding of this study is decline of HCV viral titer to a maximum of 80% with gene specific siRNAs followed by reduction in HCV protein expression. HCV replication in the Huh-7 cells was observed through detection of 5'UTR of viral copies by Real Time PCR in cells from 3^rd ^day post infection. Bian *et al. *[55] reported that 14 amino acids from the C-terminus of Core gene are required for proper function of E1 and at least 12 amino acids from C-terminus of E1 genes are required for E2 function, influencing the proper glycosylation of E1 and E2 gene. The siRNAs in the present study which showed reduction in viral titer were also tested for effect on HCV proteins expression as they are translated in a form of single polypeptide and found to be equally effective in HCV protein expression, while the effect was much greater against E2 protein expression by all siRNAs (Figure 3). This effect may be as a result of 5'UTR mediated translation inhibition and also due to the inability of interaction between 5'UTR domain III and Core during virion assembly. Our data is in agreement with Zekri *et al*. [27], who demonstrated that siRNAs against 5'UTR of HCV genotype-4 inhibited HCV replication in serum infected Huh-7 cells and Khaliq et al. [28] who tested siRNAs against HCV 3a in serum infected cells and observed the down regulation of HCV RNA. Treatment of siRNAs revealed significant inhibitory effect on HCV copy number indicating the potential of siRNAs as an efficient therapeutic strategy.

Our results demonstrate that siRNA targeting HCV 5'UTR can elicit viral RNA from infected cell and potentially offer an efficient therapeutic option for HCV infection. These results are in agreement with the previous studies which suggested that siRNA is the most efficient nucleic acid based antiviral approach that can be utilized to degrade HCV genome in the infected cells. Moreover, based on these results it is suggested that RNAi-mediated silencing of the local HCV-3a 5'UTR may be one of the important therapeutic opportunities against HCV-3a genotype. Recently Pan *et al. *[56] reported that RNAi (against 5'UTR) can be applied in combination with IFNα without effecting signal transduction to hepatocytes or interfering using RNAi which significantly enhanced their individual antiviral effects. Therefore, it can also be speculated from our pilot study that therapeutic induction of RNAi against HCV-3a 5'UTR either alone or in combination with IFN treatment might represent an alternative approach for future treatment of chronic infection.

## List of abbreviations

5'UTR: 5' untranslated region; IRES: internal ribosome entry site; HCC: Hepatocellular carcinoma; HCV: Hepatitis C virus; PEG-INF-α: pegylated interferon alpha; RNAi: RNA interference; siRNAs: small interfering RNAs

## Competing interests

The authors declare that they have no competing interests.

## Authors' contributions

SK, SJ and AP perform lab work and prepared the manuscript. UAA helped SK in lab work and literature review. SK and SJ write and critically reviewed the manuscript. SH provides all facilitates to complete this work. All authors read and approved final manuscript.

## Authors' information

Saba Khaliq (PhD Molecular Biology), Shah Jahan (PhD Molecular Biology), Asim Pervaiz (M.Phil Molecular Biology), Usman Ali Ashfaq (PhD Molecular Biology), and Sajida Hassan (PhD Molecular Biology) at CEMB, University of the Punjab, Lahore.
